# Machine learning prediction models for mortality risk in sepsis-associated acute kidney injury: evaluating early versus late CRRT initiation

**DOI:** 10.3389/fmed.2024.1483710

**Published:** 2025-01-22

**Authors:** Chuanren Zhuang, Ruomeng Hu, Ke Li, Zhengshuang Liu, Songjie Bai, Sheng Zhang, Xuehuan Wen

**Affiliations:** ^1^Department of Laboratory Medicine, Cangnan Hospital of Traditional Chinese Medicine, Wenzhou, Zhejiang, China; ^2^Department of Critical Care Medicine, Second Affiliated Hospital, Zhejiang University School of Medicine, Hangzhou, Zhejiang, China; ^3^Department of Critical Care Medicine, The People’s Hospital of Cangnan Zhejiang, Wenzhou Medical University, Wenzhou, Zhejiang, China; ^4^Department of Critical Care Medicine, Cangnan Hospital of Traditional Chinese Medicine, Wenzhou, Zhejiang, China; ^5^Department of Cardiovascular Surgery, The First Affiliated Hospital, Jiangxi Medical College, Nanchang University, Nanchang, Jiangxi, China; ^6^Department of Critical Care Medicine, Taizhou Hospital of Zhejiang Province, Wenzhou Medical University, Taizhou, China; ^7^Department of Oncology, The People’s Hospital of Cangnan Zhejiang, Wenzhou Medical University, Wenzhou, Zhejiang, China

**Keywords:** sepsis, acute kidney injury, continuous renal replacement therapy, machine learning, mortality, CRRT timing

## Abstract

**Background:**

Sepsis-associated acute kidney injury (S-AKI) has a significant impact on patient survival, with continuous renal replacement therapy (CRRT) being a crucial intervention. However, the optimal timing for CRRT initiation remains controversial.

**Methods:**

Using the MIMIC-IV database for model development and the eICU database for external validation, we analyzed patients with S-AKI to compare survival rates between early and late CRRT initiation groups. Propensity score matching was performed to address potential selection bias. Subgroup analyses stratified patients by disease severity using SOFA scores (low ≤10, medium 11–15, high >15) and creatinine levels (low ≤3 mg/dL, medium 3–5 mg/dL, high >5 mg/dL). Multiple machine learning models were developed and evaluated to predict patient prognosis, with Shapley Additive exPlanations (SHAP) analysis identifying key prognostic factors.

**Results:**

After propensity score matching, late CRRT initiation was associated with improved survival probability, but led to increased hospital and ICU stays. Subgroup analyses showed consistent trends favoring late CRRT across all SOFA categories, with the most pronounced effect in high SOFA scores (>15, *p* = 0.058). The GBM model demonstrated robust predictive performance (average C-index 0.694 in validation and test sets). SHAP analysis identified maximum lactate levels, age, and minimum SpO2 as the strongest predictors of mortality, while CRRT timing showed relatively lower impact on outcome prediction.

**Conclusion:**

While later initiation of CRRT in S-AKI patients was associated with improved survival, this benefit comes with increased healthcare resource utilization. The clinical parameters, rather than CRRT timing, are the primary determinants of patient outcomes, suggesting the need for a more personalized approach to CRRT initiation based on overall illness severity.

## Introduction

Sepsis is a life-threatening condition characterized by organ dysfunction that results from a dysregulated host response to infection (1). Among the affected organs, the kidneys are particularly vulnerable, leading to sepsis-associated acute kidney injury (S-AKI) (2). S-AKI significantly increases the risk of in-hospital mortality and long-term chronic kidney disease, exhibiting a poorer prognosis than non-septic AKI (3–5). Epidemiological studies indicate that sepsis accounts for 45–70% of all cases of AKI (6), while approximately 60% of patients with sepsis develop AKI (7).

Continuous renal replacement therapy (CRRT) has emerged as a crucial treatment modality for Patients with S-AKI due to its capacity to continuously remove toxins and regulate electrolyte and acid–base balance (3, 8). CRRT offers several advantages over conventional intermittent dialysis, including more precise volume control, improved hemodynamic stability, and more effective correction of acid–base balance and electrolyte imbalance (9). These advantages have established CRRT as the preferred renal replacement therapy for critically ill patients, as evidenced by a 2015 multinational study, which reported its use in 75.2% of AKI cases in intensive care units (ICU) (10).

Despite the widespread use of CRRT in S-AKI management, considerable debate persists regarding the optimal timing of initiation in patients lacking absolute indications. Several studies have demonstrated the benefits of early CRRT initiation, including improved survival rates and accelerated recovery of renal function (11–14). However, other studies have found no significant benefit from early initiation, and some studies have even suggested potential risks associated with the premature commencement of CRRT (15–18). This controversy primarily stems from the heterogeneity of patients with S-AKI and the limitations of current research methodologies.

Beyond the timing of CRRT initiation, many other factors also influence the prognosis of patients with S-AKI. These include patient characteristics, illness severity, and various clinical and laboratory parameters (19, 20). Given the complex interplay between these factors, there is a growing demand for prognostic models to guide personalized treatment.

In this study, we used the MIMIC-IV and EICU large-scale database to investigate the impact of CRRT initiation timing on outcomes in patients with S-AKI. Moreover, we developed and validated machine learning algorithms to predict survival in these patients, aiming to identify crucial prognostic factors influencing outcomes. By leveraging advanced analytical techniques on a large patient cohort, we aimed to offer insights that could help refine personalized and more effective management strategies for patients with S-AKI requiring CRRT.

## Methods and materials

### Data source

This retrospective study used health-related data from the MIMIC-IV (version 3.0) database, a comprehensive and widely used resource developed and maintained by the MIT Computational Physiology Laboratory. The MIMIC-IV database contains high-quality medical records of patients admitted to the ICU of Beth Israel Deaconess Medical Center (21). Data extraction was conducted by Xuehuan Wen, who adhered to all database access requirements. For external validation, we utilized the eICU Collaborative Research Database, with data access also authorized to Xuehuan Wen. As both MIMIC-IV and eICU are publicly available anonymized databases, ethical committee approval was deemed unnecessary for this study.

### Study population

The study population comprised adult patients (≥18 years) with S-AKI. Sepsis was defined according to the Third International Consensus Definitions for Sepsis and Septic Shock (Sepsis-3) criteria, requiring a Sequential Organ Failure Assessment (SOFA) score ≥ 2 points in the context of suspected or confirmed infection (1). AKI was classified using the Kidney Disease: Improving Global Outcomes (KDIGO) criteria, with inclusion requiring stage ≥1 AKI (22).

**Figure 1:**
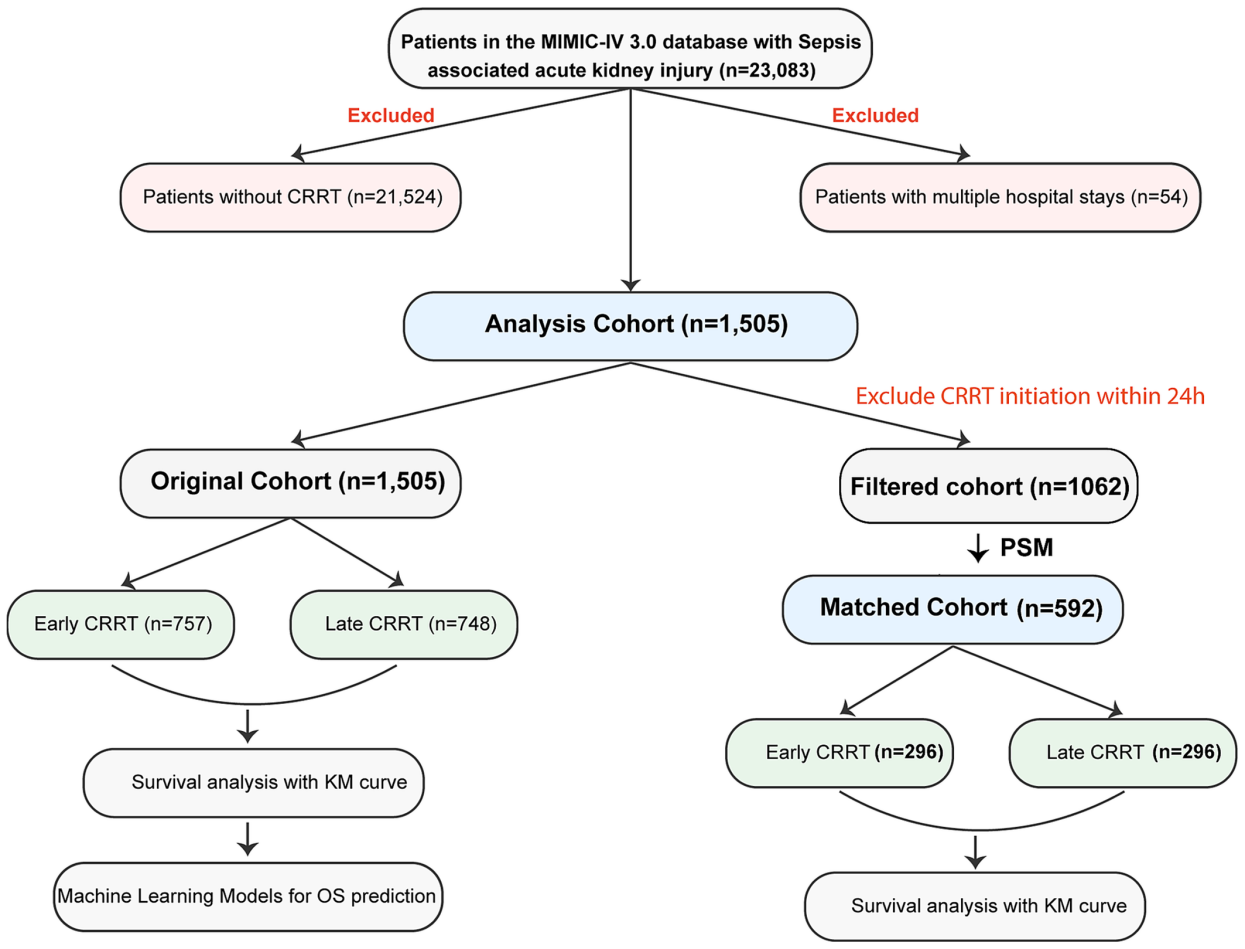
Flowchart of the study cohort. Schematic representation of patient selection methodology from the MIMIC-IV 3.0 database, delineating the sequential filtration process and subsequent analytical stratification of sepsis-associated acute kidney injury cases into original and propensity-matched cohorts for comparative outcome analysis. CRRT, continuous renal replacement therapy; PSM, propensity score matching; KM, Kaplan–Meier; OS, overall survival.

As shown in Figure 1, from an initial cohort of 23,083 patients with S-AKI, we excluded 21,524 patients who did not receive CRRT and 54 patients with multiple hospital admissions during the study period. Among the remaining 1,505 patients who received CRRT, we further excluded 443 patients who initiated CRRT within 24 h of admission to address potential confounding from mixed pre-and post-CRRT effects. This resulted in 1,062 patients for analysis. Using the median time from S-AKI onset to CRRT initiation (2.49 days) as the threshold, we stratified patients into early (≤2.49 days) and late (>2.49 days) CRRT groups. To minimize potential selection bias and confounding, we performed propensity score matching (PSM) (23) using a 1:1 nearest neighbor matching algorithm without replacement. The matching variables included disease severity indicators: SOFA score, maximum creatinine, minimum platelets, minimum mean blood pressure, maximum potassium, minimum bicarbonate, and maximum INR. The matched cohort comprised 296 patients in each group (total *n* = 592), achieving balance in these key clinical characteristics. Subsequent survival analyses were conducted using this matched cohort to minimize bias from disease severity differences between groups.

### Data collection and processing

Data extraction was executed using PostgreSQL (version 16.3.2) and Navicat Premium (version 17) with Structured Query Language (SQL) queries. This process was performed on both the MIMIC-IV and eICU databases, following identical extraction protocols to ensure consistency across datasets. The extracted variables were classified into five main groups: (1) Demographics: age, gender, race, weight, height, and BMI. (2) Comorbidities: including cardiovascular, pulmonary, hepatic, renal, and metabolic diseases. (3) Laboratory indicators: including complete blood count, metabolic panel, coagulation profile, and markers of organ function. (4) Vital signs: including blood pressure, heart rate, respiratory rate, temperature, and oxygen saturation. (5) Severity of illness scores at admission: Sequential Organ Failure Assessment (SOFA) score, which evaluates six organ systems (respiratory, coagulation, liver, cardiovascular, central nervous system, and renal) using specific clinical and laboratory parameters ranging from 0 (normal) to 4 (most abnormal) for each system.

The feature set used for analysis comprised three main components: clinical parameters collected within the first 24 h of ICU admission, patient comorbidities, and the time interval from AKI diagnosis to CRRT initiation. The baseline clinical parameters included demographic information, SOFA scores and their components, laboratory results, and vital signs. To ensure data integrity, we employed a systematic approach to handle missing data. Patient records with more than 20% missing features were excluded from the analysis. For the remaining records, features with missing values exceeding 10% were removed from the dataset. The remaining missing values were imputed using the Multiple Imputation by Chained Equations (MICE) method, implemented through the miceR package in R. This comprehensive approach to data collection and preprocessing ensured a robust foundation for subsequent analyses while minimizing potential biases from missing data.

### Propensity score matching analysis

To minimize selection bias between early and late CRRT groups, we performed PSM using the “MatchIt” package in R (24). The matching model incorporated six key clinical parameters that typically influence CRRT initiation decisions: SOFA score (reflecting overall illness severity), maximum creatinine (indicating kidney dysfunction), minimum mean blood pressure (hemodynamic status), maximum potassium (electrolyte derangement), minimum bicarbonate (metabolic acidosis), and maximum INR (coagulation status). These variables were selected based on their clinical relevance to CRRT timing decisions in critical care settings.

We employed nearest-neighbor matching with customized parameters to ensure precise matching on these critical variables. The matching was performed with a 1:1 ratio, and balance assessment of covariates before and after matching was conducted using standardized mean differences, with values less than 0.1 indicating adequate balance. The quality of matching was further evaluated through visual inspection of propensity score distributions and covariate balance plots.

### Clinical outcomes

The primary endpoint of this study was all-cause mortality. We analyzed survival status at multiple time points, including short-term outcomes at 14, 28, and 90 days, as well as long-term outcomes at 1, 2, and 3 years. Among these, 28-day mortality and survival time were designated as key indicators and utilized for subsequent predictive model development.

### Survival analysis with Kaplan–Meier curves

We conducted survival analyses on both the original cohort and the propensity score-matched cohort using the Kaplan–Meier method consistent with our previous work (25). Using the median time interval between AKI onset and CRRT initiation as the threshold, patients were stratified into early and late CRRT groups. The analysis was performed using R (version 4.3.2) and encompassed both short-term outcomes (14-day, 28-day, and 90-day survival) and long-term outcomes (1-, 2-, and 3-year survival). We employed the log-rank test to assess statistical differences in survival distributions between groups. To ensure robustness of our findings, we performed parallel analyses in both the MIMIC-IV and eICU databases. The initial analysis on the complete cohort examined unadjusted survival differences, while the subsequent analysis on the propensity score-matched cohorts provided survival outcomes with balanced baseline characteristics, thus minimizing potential confounding effects.

### Construction of multiple machine learning models

We developed and validated multiple machine learning models using features collected within the first 24 h of ICU admission, as detailed in Table 1. The dataset was randomly split into training (70%) and internal validation (30%) sets, with external validation performed using the eICU database. To ensure robust predictive performance, we implemented multiple machine learning algorithms (26), including: Gradient Boosting Machine (GBM, an ensemble learning technique that sequentially builds decision trees to improve predictive accuracy by learning from previous errors), Random Survival Forest (RSF, which combines multiple decision trees to analyze time-to-event data), and various regression-based approaches including LASSO (Least Absolute Shrinkage and Selection Operator), Ridge regression, and Elastic Net (Enet) with *α* values ranging from 0.1 to 0.9.

**Table 1:** Baseline characteristics of patients with sepsis-associated AKI stratified by time from AKI onset to CRRT initiation (parameters from first 24 hours of ICU admission).

Variables	Total (*n* = 1,505)	Late (*n* = 748)	Early (*n* = 757)	*p*
AKI to CRRT days	1.50 [0.36, 3.75]	3.76 [2.39, 7.02]	0.36 [0.00, 0.78]	<0.001
CRRT mode				
CVVH	20 (1.3)	16 (2.1)	4 (0.5)	
CVVHD	11 (0.7)	4 (0.5)	7 (0.9)	
CVVHDF	622 (41.3)	295 (39.4)	327 (43.2)	
SCUF	1 (0.1)	1 (0.1)	0 (0.0)	
Age	64.11 [53.90, 74.19]	66.02 [55.37, 75.17]	62.88 [52.41, 72.42]	<0.001
Gender				
Female	608 (40.4)	291 (38.9)	317 (41.9)	0.262
Male	897 (59.6)	457 (61.1)	440 (58.1)
Race				
ASIAN	40 (2.7)	22 (2.9)	18 (2.4)	0.162
BLACK	190 (12.6)	89 (11.9)	101 (13.3)
WHITE	857 (56.9)	445 (59.5)	412 (54.4)
OTHER	418 (27.8)	192 (25.7)	226 (29.9)
Height (cm)	170.18 [163.00, 178.00]	170.18 [163.00, 178.00]	170.18 [163.00, 178.00]	0.388
Weight (kg)	88.34 [74.50, 104.83]	88.44 [75.28, 105.07]	88.00 [73.91, 104.31]	0.469
BMI	30.57 [26.18, 35.66]	30.80 [26.68, 35.44]	30.41 [25.95, 36.10]	0.343
Comorbidities				
Myocardial Infarct (+) *n* (%)	306 (20.3)	149 (19.9)	157 (20.7)	0.741
Congestive Heart Failure (+) *n* (%)	562 (37.3)	266 (35.6)	296 (39.1)	0.172
Peripheral Vascular Disease (+) *n* (%)	241 (16.0)	116 (15.5)	125 (16.5)	0.645
Cerebrovascular Disease (+) *n* (%)	174 (11.6)	105 (14.0)	69 (9.1)	0.004
Dementia (+) *n* (%)	27 (1.8)	15 (2.0)	12 (1.6)	0.675
Chronic Pulmonary Disease (+) *n* (%)	388 (25.8)	196 (26.2)	192 (25.4)	0.754
Rheumatic Disease (+) *n* (%)	55 (3.7)	24 (3.2)	31 (4.1)	0.436
Peptic Ulcer Disease (+) *n* (%)	68 (4.5)	33 (4.4)	35 (4.6)	0.941
Mild Liver Disease (+) *n* (%)	440 (29.2)	212 (28.3)	228 (30.1)	0.483
Diabetes without Complication (+), *n* (%)	413 (27.4)	213 (28.5)	200 (26.4)	0.403
Diabetes with Complication (+), *n* (%)	275 (18.3)	120 (16.0)	155 (20.5)	0.031
Paraplegia (+) *n* (%)	27 (1.8)	15 (2.0)	12 (1.6)	0.675
Renal Disease (+) *n* (%)	656 (43.6)	314 (42.0)	342 (45.2)	0.230
Malignant Cancer (+) *n* (%)	141 (9.4)	78 (10.4)	63 (8.3)	0.189
Severe Liver Disease (+) *n* (%)	221 (14.7)	112 (15.0)	109 (14.4)	0.809
Metastatic Solid Tumor (+) *n* (%)	36 (2.4)	17 (2.3)	19 (2.5)	0.895
AIDS (+) *n* (%)	9 (0.6)	3 (0.4)	6 (0.8)	0.515
Vasoactive agents (+), *n* (%)	1,186 (78.8)	600 (80.2)	586 (77.4)	0.205
Invasive ventilation (+), *n* (%)	1,342 (89.2)	689 (92.1)	653 (86.3)	<0.001
28-day mortality (+), *n* (%)	750 (49.8)	362 (48.4)	388 (51.3)	0.290
Laboratory tests				
Lactate min (mmol/L)	1.80 [1.20, 3.00]	1.60 [1.10, 2.40]	2.10 [1.30, 4.00]	<0.001
Lactate max (mmol/L)	3.70 [2.00, 7.90]	3.00 [1.80, 5.20]	5.20 [2.30, 10.50]	<0.001
PH min	7.22 [7.12, 7.31]	7.27 [7.19, 7.34]	7.17 [7.06, 7.26]	<0.001
PH max	7.39 [7.32, 7.44]	7.41 [7.35, 7.46]	7.37 [7.30, 7.43]	<0.001
Base excess min (mEq/L)	−8.00 [−14.00, −3.00]	−6.00 [−10.00, −1.00]	−11.00 [−17.00, −6.00]	<0.001
Base excess max (mEq/L)	−1.00 [−5.00, 1.00]	0.00 [−3.00, 2.00]	−2.00 [−6.00, 0.00]	<0.001
Wbc min (K/uL)	10.80 [6.70, 15.50]	10.50 [6.70, 14.75]	11.00 [6.68, 16.30]	0.064
Bicarbonate min (mEq/L)	22.00 [19.00, 25.00]	23.00 [20.00, 26.00]	21.00 [18.00, 24.00]	<0.001
Bicarbonate max (mEq/L)	17.00 [13.00, 21.00]	19.00 [16.00, 22.00]	15.00 [11.00, 20.00]	<0.001
Wbc max (K/uL)	16.00 [10.90, 22.75]	15.20 [10.55, 20.50]	17.30 [11.38, 24.72]	<0.001
Abs Basophils min (K/uL)	0.01 [0.00, 0.03]	0.01 [0.00, 0.03]	0.01 [0.00, 0.03]	0.092
Abs Basophils max (K/uL)	0.02 [0.00, 0.05]	0.02 [0.00, 0.05]	0.02 [0.00, 0.05]	0.409
Abs Eosinophils min (K/uL)	0.01 [0.00, 0.07]	0.01 [0.00, 0.09]	0.00 [0.00, 0.06]	0.137
Abs Eosinophils max (K/uL)	0.02 [0.00, 0.12]	0.03 [0.00, 0.13]	0.02 [0.00, 0.11]	0.187
Abs Lymphocytes min (K/uL)	0.82 [0.46, 1.38]	0.82 [0.46, 1.34]	0.81 [0.46, 1.40]	0.945
Abs Lymphocytes max (K/uL)	0.98 [0.59, 1.71]	0.96 [0.57, 1.66]	1.00 [0.61, 1.72]	0.324
Abs Monocytes min (K/uL)	0.50 [0.23, 0.92]	0.49 [0.24, 0.91]	0.52 [0.22, 0.93]	0.542
Abs Monocytes max (K/uL)	0.66 [0.37, 1.09]	0.64 [0.36, 1.04]	0.68 [0.37, 1.16]	0.226
Abs Neutrophils min (K/uL)	10.09 [6.01, 15.94]	9.86 [5.83, 14.19]	10.58 [6.27, 16.92]	0.041
Abs Neutrophils max (K/uL)	11.43 [7.22, 17.48]	11.05 [6.96, 16.35]	11.81 [7.40, 19.06]	0.004
Calcium min (mEq/L) (K/uL)	7.70 [7.10, 8.40]	7.85 [7.20, 8.40]	7.60 [6.90, 8.30]	<0.001
Calcium max (mEq/L) (K/uL)	8.60 [8.00, 9.20]	8.50 [7.90, 9.00]	8.70 [8.10, 9.40]	<0.001
Chloride min (mEq/L)	99.00 [94.00, 104.00]	100.00 [95.00, 105.00]	97.00 [93.00, 102.00]	<0.001
Chloride max (mEq/L)	104.00 [99.00, 108.00]	105.00 [100.00, 109.00]	103.00 [98.00, 108.00]	<0.001
Sodium min (mEq/L)	135.00 [132.00, 139.00]	136.00 [132.00, 139.00]	135.00 [131.00, 138.00]	<0.001
Sodium max (mEq/L)	139.00 [136.00, 143.00]	139.00 [136.00, 143.00]	139.00 [135.75, 143.00]	0.848
Potassium min (mEq/L)	4.10 [3.60, 4.50]	4.00 [3.50, 4.50]	4.10 [3.60, 4.60]	0.001
Potassium max (mEq/L)	5.00 [4.40, 5.80]	4.70 [4.30, 5.40]	5.30 [4.60, 6.20]	<0.001
Inr min	1.40 [1.20, 1.80]	1.30 [1.12, 1.60]	1.40 [1.20, 1.90]	<0.001
Inr max	1.60 [1.30, 2.40]	1.50 [1.30, 2.10]	1.80 [1.30, 2.95]	<0.001
Pt min (s)	15.00 [12.90, 19.30]	14.50 [12.70, 18.00]	15.60 [13.20, 20.70]	<0.001
Pt max (s)	17.90 [14.30, 26.50]	16.80 [13.90, 22.87]	19.40 [14.90, 31.80]	<0.001
Ptt min (s)	32.30 [28.15, 39.30]	31.90 [27.80, 38.85]	32.70 [28.60, 39.50]	0.076
Ptt max (s)	42.60 [33.10, 68.25]	40.85 [32.40, 63.12]	45.10 [33.50, 74.65]	0.002
Platelet min (K/uL)	135.00 [71.00, 206.00]	140.00 [82.00, 203.00]	125.00 [64.00, 208.00]	0.029
Platelet max (K/uL)	186.00 [122.00, 264.50]	184.00 [124.50, 253.00]	187.50 [118.00, 278.25]	0.455
Hematocrit min (%)	27.40 [23.10, 32.90]	27.20 [23.10, 32.75]	27.60 [22.90, 33.00]	0.867
Hematocrit max (%)	33.20 [28.70, 38.30]	32.80 [28.60, 37.75]	33.60 [28.78, 38.70]	0.143
Hemoglobin min (g/dL)	8.90 [7.50, 10.60]	8.90 [7.60, 10.60]	8.90 [7.40, 10.55]	0.369
Hemoglobin max (g/dL)	10.70 [9.20, 12.30]	10.60 [9.20, 12.30]	10.70 [9.20, 12.35]	0.561
CRP min (mg/L)	131.70 [67.20, 202.00]	120.80 [67.05, 198.88]	142.50 [69.90, 204.50]	0.520
CRP max (mg/L)	134.40 [68.70, 210.00]	128.35 [68.35, 213.12]	158.30 [69.90, 205.40]	0.584
ALT Min (IU/L)	37.00 [20.00, 120.50]	35.00 [20.00, 89.00]	42.00 [20.00, 193.25]	0.003
ALT Max (IU/L)	52.00 [23.00, 249.50]	43.00 [23.00, 121.00]	66.50 [24.00, 535.25]	<0.001
AST Min (IU/L)	72.00 [34.00, 235.75]	66.50 [32.00, 161.75]	79.50 [37.75, 408.25]	<0.001
AST Max (IU/L)	108.00 [42.00, 509.50]	84.00 [39.00, 241.75]	137.50 [48.00, 1133.00]	<0.001
Bilirubin Min (mg/dL)	0.90 [0.40, 2.60]	0.90 [0.40, 2.70]	0.80 [0.40, 2.50]	0.851
Bilirubin Max (mg/dL)	1.20 [0.50, 3.40]	1.10 [0.50, 3.50]	1.30 [0.50, 3.40]	0.536
LDH Min (IU/L)	444.00 [281.00, 820.25]	402.00 [273.00, 709.00]	476.00 [299.00, 977.00]	<0.001
LDH Max (IU/L)	529.50 [308, 1,129]	456.00 [299.00, 844.00]	618.00 [336, 1,781.00]	<0.001
Albumin Min (g/dL)	2.80 [2.20, 3.20]	2.70 [2.30, 3.20]	2.80 [2.20, 3.30]	0.806
Albumin Max (g/dL)	2.95 [2.50, 3.40]	2.90 [2.50, 3.40]	3.00 [2.50, 3.40]	0.576
Bun min (mg/dL)	33.00 [21.00, 53.00]	30.00 [19.00, 50.50]	36.00 [22.00, 55.00]	0.002
Bun max (mg/dL)	44.00 [28.00, 68.00]	38.00 [25.00, 60.50]	49.00 [32.00, 74.25]	<0.001
Creatinine Min (mg/dL)	2.20 [1.30, 3.60]	1.70 [1.10, 3.00]	2.70 [1.70, 4.20]	<0.001
Creatinine Max (mg/dL)	3.00 [1.90, 4.80]	2.30 [1.40, 3.70]	3.90 [2.48, 5.80]	<0.001
Urine output				
Urine output (mL)	490.00 [140, 1,183.00]	820.00 [305, 1,598.75]	234.50 [66.50, 720.75]	<0.001
Severity scores				
SOFA	10.00 [8.00, 13.00]	10.00 [7.00, 12.00]	12.00 [9.00, 14.00]	<0.001
Coagulation	1.00 [0.00, 2.00]	1.00 [0.00, 2.00]	1.00 [0.00, 2.00]	0.006
Liver	1.00 [0.00, 2.00]	0.00 [0.00, 2.00]	1.00 [0.00, 2.00]	0.222
CNS	0.00 [0.00, 1.00]	0.00 [0.00, 1.00]	0.00 [0.00, 1.00]	0.163
Renal	3.00 [2.00, 4.00]	2.00 [1.00, 3.00]	4.00 [3.00, 4.00]	<0.001
Cardiovascular	3.00 [1.00, 4.00]	3.00 [1.00, 4.00]	4.00 [1.00, 4.00]	<0.001
Respiration	3.00 [2.00, 4.00]	3.00 [2.00, 4.00]	3.00 [3.00, 4.00]	<0.001
Vital signs				
Heart Rate min (beats/min)	76.00 [63.00, 87.00]	76.00 [65.00, 87.00]	75.00 [62.00, 88.00]	0.185
Heart Rate max (beats/min)	111.00 [95.00, 128.00]	109.00 [95.00, 125.00]	113.00 [96.00, 130.00]	0.012
Systolic BP min (mmHg)	81.00 [72.00, 90.00]	83.00 [75.00, 91.12]	79.00 [69.00, 90.00]	<0.001
Systolic BP max (mmHg)	142.00 [129.00, 158.00]	144.00 [129.00, 158.25]	141.00 [128.00, 157.00]	0.144
Diastolic BP min (mmHg)	42.00 [35.00, 48.00]	43.00 [36.00, 49.00]	41.00 [34.00, 47.00]	<0.001
Diastolic BP max (mmHg)	82.00 [71.00, 96.00]	83.00 [72.00, 97.00]	81.00 [70.00, 94.00]	0.021
Mean BP min (mmHg)	54.00 [46.00, 61.00]	55.00 [48.00, 62.00]	53.00 [43.00, 60.00]	<0.001
Mean BP max (mmHg)	99.00 [89.00, 113.00]	100.00 [90.00, 114.00]	99.00 [89.00, 113.00]	0.182
Respiratory Rate min (beats/min)	13.00 [10.00, 16.00]	13.00 [10.00, 16.00]	13.00 [10.00, 16.00]	0.598
Respiratory Rate max (beats/min)	30.00 [26.00, 34.00]	30.00 [26.00, 34.00]	30.00 [26.00, 35.00]	0.022
Temperature min (°C)	36.33 [35.56, 36.61]	36.44 [35.89, 36.72]	36.11 [35.17, 36.50]	<0.001
Temperature max (°C)	37.33 [36.90, 37.94]	37.36 [36.94, 38.06]	37.28 [36.89, 37.89]	0.022
SpO2 min (%)	91.00 [87.00, 94.00]	91.00 [88.00, 94.00]	90.00 [85.00, 94.00]	<0.001
SpO2 max (%)	100.00 [100.00, 100.00]	100.00 [99.00, 100.00]	100.00 [100.00, 100.00]	0.046
Glucose min (mg/dL)	111.00 [87.00, 143.00]	115.00 [92.00, 145.00]	107.00 [82.00, 141.25]	<0.001
Glucose max (mg/dL)	176.50 [135.00, 252.00]	166.00 [129.00, 227.00]	191.50 [142.00, 269.25]	<0.001

Additional algorithms included COXboost (an algorithm that enhances standard Cox regression through boosting), Partial Least Squares Regression for Cox models (plsRcox, which handles high-dimensional data while maintaining interpretability), Supervised Principal Components (superPC, which identifies relevant feature combinations for survival prediction), and Survival Support Vector Machine (SVM, which optimizes prediction boundaries for survival outcomes).

Furthermore, all numeric characteristics were initially categorized through discrete code, given the considerable discrepancy in their values. These diverse algorithms were selected to comprehensively explore various modeling approaches, enabling us to identify the most effective and robust predictive model for our specific dataset and research objectives.

### Validation of model with survival ROC

To validate the GBM model, we conducted a survival receiver operating characteristic (ROC) analysis in the validation dataset. The analysis was conducted using the ‘survivalROC’ package in R (27). Time-dependent ROC curves were generated to assess the model’s discriminative ability at two clinically relevant timepoints: 14-day and 28-day mortality. The analysis was systematically performed across three distinct datasets: the training set (used for model development), the internal validation set (for initial performance verification), and the external test set (using eICU data for independent validation). For each timepoint and dataset, we calculated the area under the ROC curve (AUC) to quantify the model’s discriminative capability.

### SHAP analysis for feature importance

To enhance the interpretability of our GBM model and understand the relative contribution of each clinical feature to mortality prediction, we employed Shapley Additive exPlanations (SHAP) analysis. SHAP values, based on cooperative game theory principles, quantify how each feature influences individual predictions by comparing model outputs with and without that feature present. We calculated SHAP values for each patient case using the ‘iml’ R package (28). The results were visualized using the ‘shapviz’ R package, which generated comprehensive plots showing both global feature importance and local feature effects.

## Results

### Baseline characteristics

A total of 1,505 patients were enrolled in this study. The median age of the participants was 64.11 years (interquartile range [IQR]: 53.9–74.19), and 897 (59.6%) were male. The median time from AKI onset to CRRT initiation was 1.5 days (IQR: 0.36–3.75). CRRT modes were recorded for 654 patients, with continuous venovenous hemodiafiltration (CVVHDF) being the most prevalent (*n* = 622, 95.1%). The 28-day mortality rate was 49.8%.

Patients were stratified into two groups: Early (≤1.5 days) and Late (>1.5 days) initiation, based on the median time from the onset of AKI to the start of CRRT (1.5 days). Into Early (≤1.5 days) and Late (>1.5 days) initiation groups based on the median time from AKI onset to CRRT initiation (1.5 days). Baseline characteristics derived from first-day measurements were both significant and non-significant differences between groups (Table 1). Compared to the early initiation group, the Late initiation group demonstrated a higher prevalence of cerebrovascular disease (14% vs. 9.1%, *p* = 0.004) and invasive ventilation requirements (92.1% vs. 86.3%, *p* < 0.001). However, this group exhibited lower disease severity markers, including maximum creatinine (2.3 vs. 3.9 mg/dL), SOFA score (10 vs. 12), and lactate levels (3.0 vs. 5.2 mmol/L). These differences in baseline characteristics suggested less severe illness in the Late initiation group, highlighting the need for propensity score matching in subsequent analyses.

### Survival outcomes stratified by CRRT initiation timing

Initial analysis of 28-day mortality rates between early and late CRRT initiation groups (51.3% versus 48.4%) revealed no significant difference in crude mortality (*p* = 0.29). However, detailed temporal analysis through Kaplan–Meier survival curves demonstrated substantial differences in survival trajectories, particularly during the early follow-up period.

In the original cohort (*n* = 1,505; 748 late vs. 757 early), the late initiation group demonstrated significantly better survival across all time horizons. Short-term analyses revealed superior survival in the late group at 14 days (*p* < 0.0001, Supplementary Figure S1A), 28 days (*p* = 0.00051, Supplementary Figure S1B), and 90 days (*p* = 0.0042, Supplementary Figure S1C), with the most pronounced difference observed within the first 28 days. This survival advantage persisted in long-term follow-up, with significantly better outcomes in the late initiation group at 12 months (*p* = 0.004, Supplementary Figure S1D), 24 months (*p* = 0.0089, Supplementary Figure S1E), and 36 months (*p* = 0.0084, Supplementary Figure S1F).

**Figure 2:**
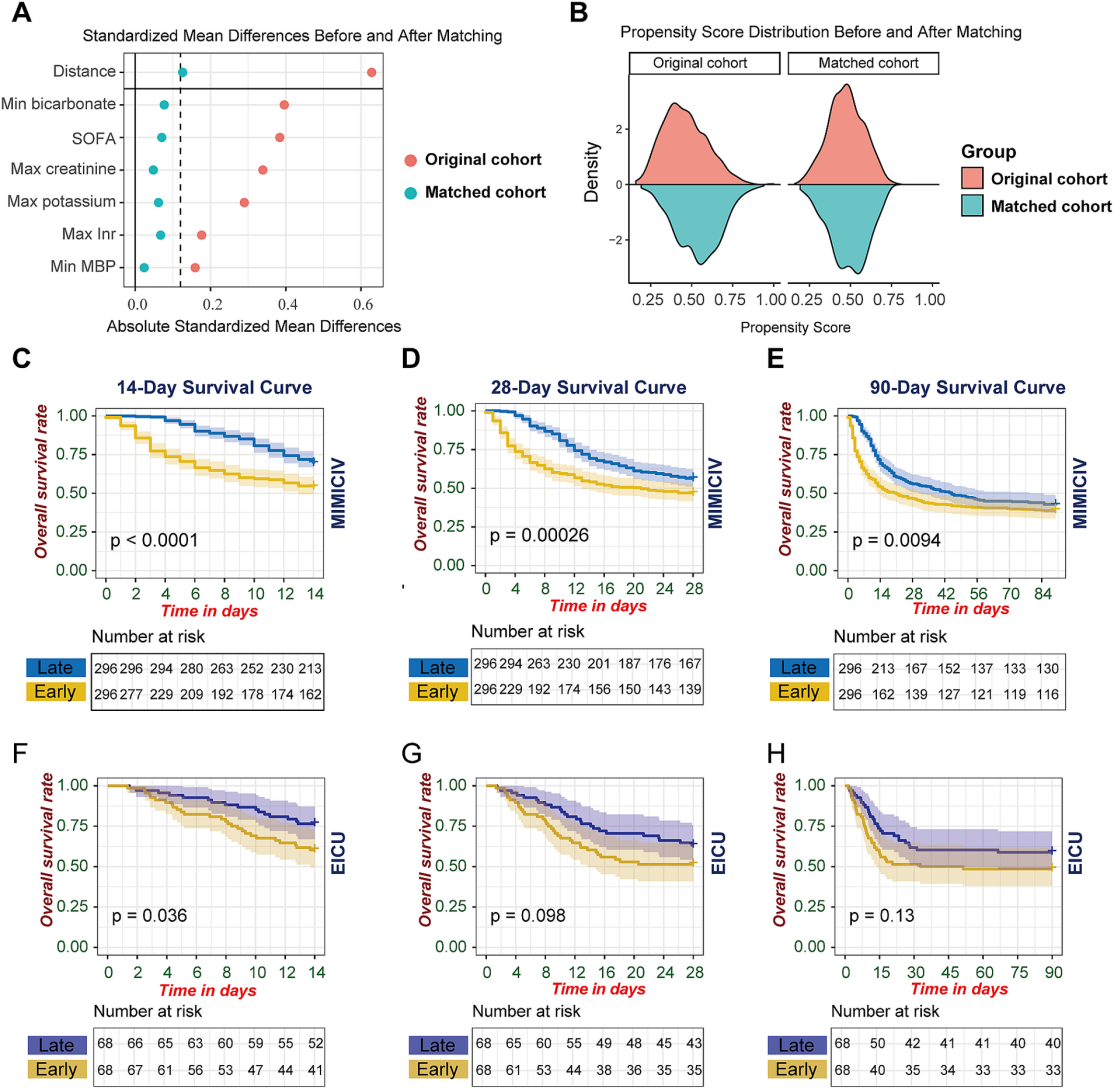
Propensity score matching analysis and survival outcomes stratified by CRRT initiation timing. **(A)** Standardized mean differences of baseline covariates pre-and post-propensity score matching. **(B)** Propensity score distribution in early versus late CRRT cohorts before and after matching. **(C–H)** Kaplan–Meier survival estimates comparing early versus late CRRT initiation: MIMIC-IV cohort at **(C)** 14 days, **(D)** 28 days, and **(E)** 90 days; eICU cohort at **(F)** 14 days, **(G)** 28 days, and **(H)** 90 days post-CRRT initiation. Early versus late CRRT initiation was dichotomized at the median time interval from AKI onset to CRRT initiation. Shaded areas represent 95% confidence intervals. Log-rank test *p* values compare survival distributions between groups. At-risk tables display the number of patients under observation at specified time points.

To enhance the validity of our analysis, we first excluded patients who received CRRT within 24 h of ICU admission. This important methodological decision was made to eliminate potential confounding effects, as baseline clinical parameters collected during the first 24 h would be influenced by the CRRT intervention itself in these early-initiation cases. After this exclusion, we performed propensity score matching incorporating six key clinical parameters: SOFA score, maximum creatinine, minimum mean blood pressure, maximum potassium, minimum bicarbonate, and maximum INR. Post-matching analysis demonstrated excellent covariate balance, with standardized mean differences (SMD) reduced to below 0.1 for all variables (Figure 2A). The density distribution of propensity scores showed marked improvement in overlap between groups after matching (Figure 2B). The non-significant differences in baseline characteristics between groups after matching, as demonstrated by *p* values in Supplementary Tables S1, S2, further validated the successful balancing of covariates.

In the MIMIC-IV matched cohort analysis, the survival advantage of late CRRT initiation was evident across multiple time horizons. Short-term survival analysis revealed significant differences at 14 days (*p* < 0.0001), 28 days (*p* = 0.00026), and 90 days (*p* = 0.0094) (Figures 2C–E). The survival benefit was most pronounced in the early period, particularly within the first 14 days post-initiation.

To validate these findings, we performed parallel analyses in the eICU database. The external validation demonstrated a consistent trend, though with varying levels of statistical significance. The late CRRT group showed better survival at 14 days (*p* = 0.036, Figure 2F), while differences at 28 days (*p* = 0.098, Figure 2G) and 90 days (*p* = 0.13, Figure 2H) did not reach statistical significance (Figures 2F–H). The consistency of the survival pattern across both databases, particularly in the early period, strengthens the evidence supporting the potential benefit of later CRRT initiation in patients with S-AKI.

### Subgroup and clinical outcomes analysis

**Figure 3:**
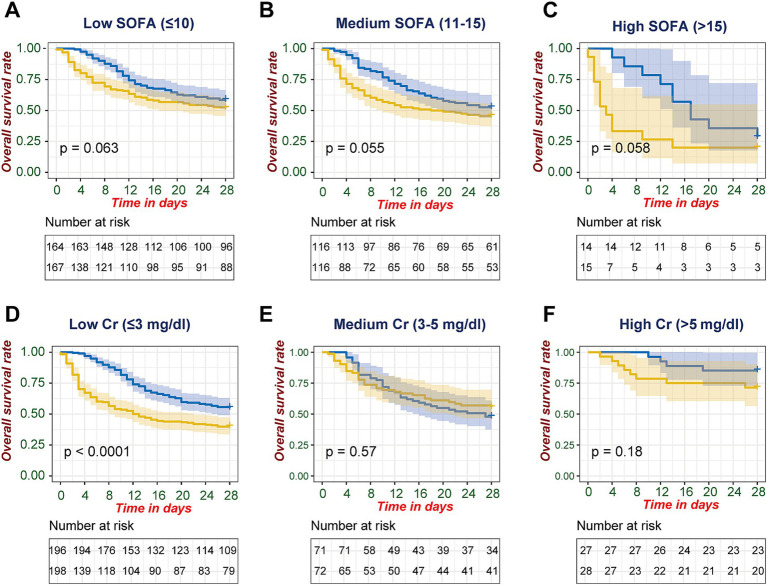
Stratified analysis of 28-day survival outcomes by SOFA score and creatinine levels. Kaplan–Meier survival analyses comparing early versus late CRRT initiation stratified by disease severity markers. **(A–C)** SOFA score stratification: **(A)** Low SOFA (≤10), **(B)** Medium SOFA (11–15), and **(C)** High SOFA (>15). **(D–F)** First-day maximum serum creatinine stratification: **(D)** Low Cr (≤3 mg/dL), **(E)** Medium Cr (3–5 mg/dL), and **(F)** High Cr (>5 mg/dL). Late and early CRRT initiation groups are represented by blue and yellow curves, respectively. Shaded areas indicate 95% confidence intervals. Log-rank test *p*-values are shown for between-group comparisons. Numbers at risk are displayed below each curve at corresponding time points.

To further investigate the relationship between CRRT timing and survival in different patient subgroups, we stratified patients based on disease severity (SOFA score) and kidney injury severity (maximum creatinine levels). In SOFA score-based stratification, although not reaching conventional statistical significance, the Kaplan–Meier curves demonstrated consistent separation favoring late CRRT across all severity categories. This survival advantage was observed in patients with low SOFA scores (≤10, *p* = 0.063, Figure 3A) and medium SOFA groups (11–15, *p* = 0.055, Figure 3B), and was most pronounced in patients with high SOFA scores (>15, *p* = 0.058, Figure 3C), where the curves showed the widest separation, with early CRRT associated with notably lower survival rates by day 28.

When stratified by maximum day-1 creatinine levels, a significant survival advantage for late CRRT was observed in the low creatinine group (≤3 mg/dL, *p* < 0.0001, Figure 3D), while no significant differences were found in medium (3–5 mg/dL, *p* = 0.57, Figure 3E) or high creatinine groups (>5 mg/dL, *p* = 0.18, Figure 3F).

To evaluate potential complications and clinical implications of different CRRT timing strategies, we analyzed hemodynamic stability and bleeding risk. Temporal analysis of clinical parameters revealed distinct patterns between early and late CRRT groups. Mean arterial pressure remained stable in both groups over the 7-day observation period, with the late CRRT group maintaining slightly higher values (74.7 ± 10.6 vs. 73.7 ± 11.1 mmHg, *p* = 0.043, Supplementary Figure S2A). Hemoglobin levels showed a progressive decline in both groups after day 2, with the early CRRT group starting from a higher baseline (9.3 ± 1.5 vs. 8.9 ± 1.4 g/dL, *p* = 0.004, Supplementary Figure S2B; Supplementary Table S3).

Given the potential impact of CRRT timing on healthcare resource utilization, we further examined clinical outcomes and hospital course metrics. Analysis revealed significantly longer durations in the late CRRT group across multiple parameters. These included hospital length of stay (median difference 5.2 days, *p* < 0.001), ICU length of stay (median difference 4.8 days, *p* < 0.001), and mechanical ventilation duration (median difference 3.9 days, *p* < 0.001). The consistent pattern of increased resource utilization in the late CRRT group suggests a complex relationship between intervention timing and recovery trajectory (Supplementary Figure S2C).

### Establishment of multiple machine learning methods for predicting overall survival of patients with S-AKI

**Figure 4:**
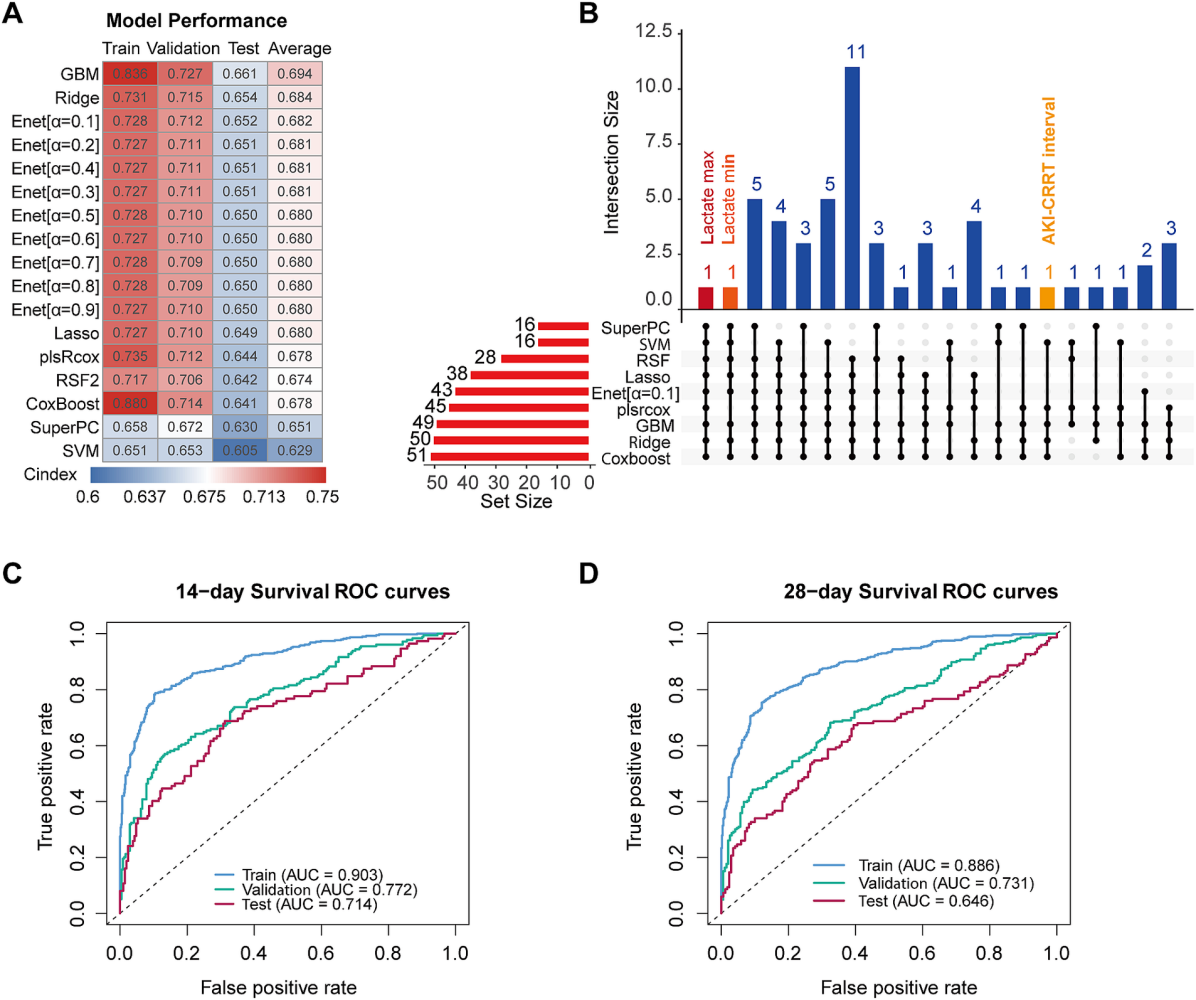
Machine learning model construction and performance analysis. **(A)** C-index heatmap comparing model performance across training, validation, and test datasets, with mean C-index values displayed. **(B)** UpSet plot illustrating the intersection of key model features with occurrence frequency greater than 3. **(C)** Time-dependent ROC curves for the GBM model at 14 days across training, validation, and test datasets. **(D)** Time-dependent ROC curves for the GBM model at 28 days across training, validation, and test datasets.

Following the establishment of a significant association between CRRT initiation timing and overall survival in patients with S-AKI, we developed a comprehensive predictive framework using machine learning techniques. The MIMIC-IV cohort was divided into training and validation sets, while the eICU database served as an external test set to evaluate model generalizability. Using the features outlined in the Methods section, we implemented and evaluated 17 different machine learning models. The performance of each model was assessed using the C-index across training, validation, and external test sets (Figure 4A). The GBM model demonstrated the strongest performance with an average C-index of 0.694 between validation and external test sets.

We then analyzed feature selection patterns across models using an upset plot, selecting Elastic Net (*α* = 0.1) as the representative Elastic Net model (Figure 4B). The intersection analysis revealed Lactate max as the only feature consistently selected across all models. Notably, the time from AKI onset to CRRT initiation appeared in five different models, suggesting its potential predictive value.

To rigorously evaluate the model’s predictive performance, we conducted survival ROC analyses at clinically relevant time points across all three datasets. The 14-day survival predictions showed AUCs of 0.903, 0.772, and 0.714 for the training, validation, and test sets, respectively (Figure 4C). Similarly, for 28-day survival, the model achieved AUCs of 0.886, 0.731, and 0.646 (Figure 4D).

### Identification of key prognostic features in patients with S-AKI

**Figure 5:**
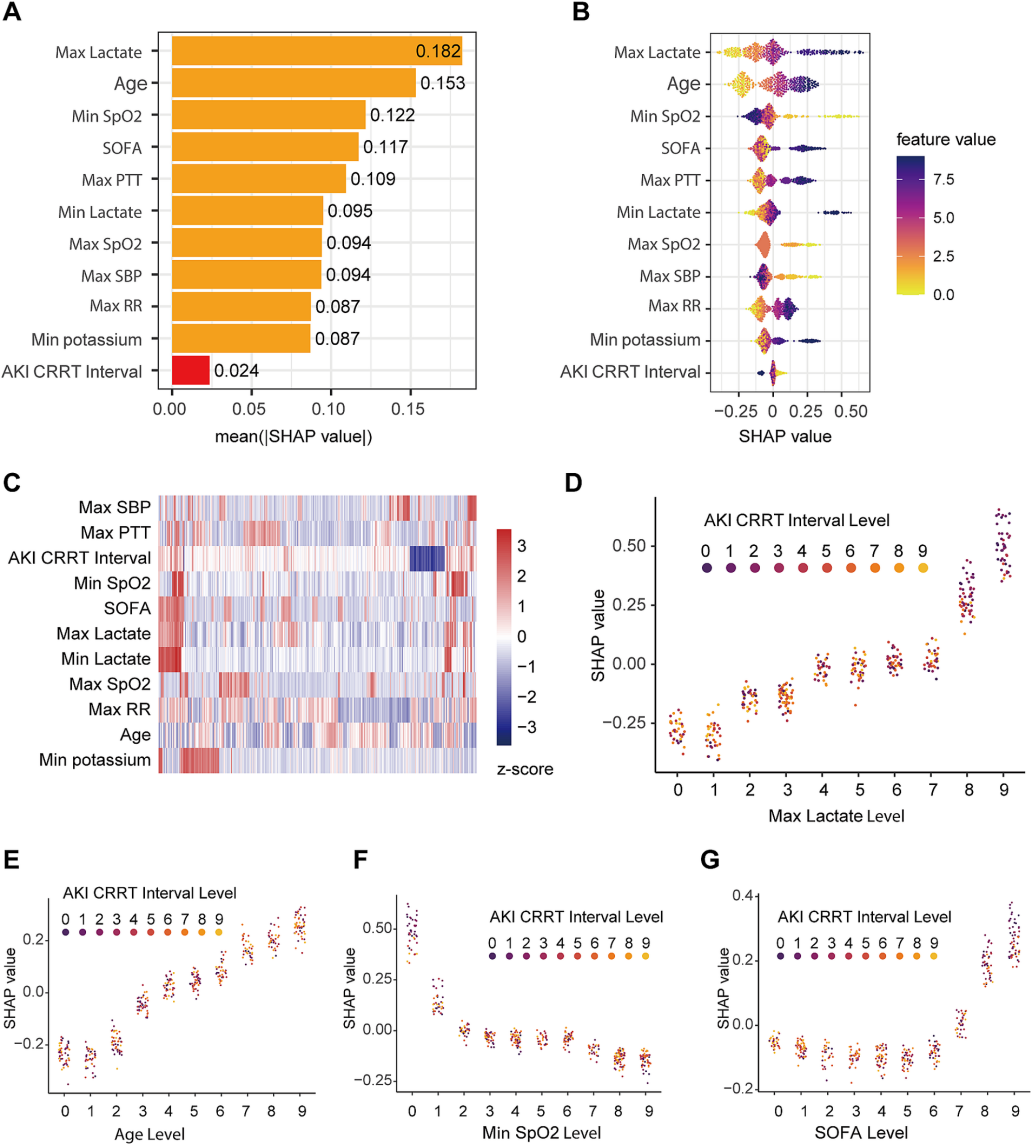
SHAP value analysis of features in the validation dataset. **(A)** Ranking of feature importance based on mean absolute SHAP values, showing top 10 clinical parameters and AKI-to-CRRT interval. **(B)** SHAP value distribution for key features with color gradient indicating feature values; points represent individual cases. **(C)** Feature value heatmap showing standardized (z-score) distribution across the validation cohort. **(D–G)** SHAP value interaction plots demonstrating the relationship between AKI-to-CRRT interval and: **(D)** maximum lactate levels, **(E)** age, **(F)** minimum SpO2, and **(G)** SOFA score. Color gradients represent AKI-to-CRRT interval levels (0–9).

To identify vital factors influencing prognosis in S-AKI patients, we evaluated feature importance within the GBM model using SHAP values. Figure 5A illustrates the mean SHAP values for the top 10 features and the duration from AKI onset to CRRT initiation (AKI CRRT Interval), ranked by their impact on prediction. Maximum lactate emerged as the most influential factor (mean SHAP value: 0.182), followed by age (0.153), minimum SpO2 (0.122), and SOFA score (0.117). Notably, the AKI CRRT Interval showed the smallest impact (mean SHAP value: 0.024) among all analyzed features.

The beeswarm plot (Figure 5B) visualized the distribution of both feature values and their corresponding SHAP values across the patient cohort. Higher maximum lactate values (shown in purple) were associated with higher SHAP values, indicating increased mortality risk. Age and minimum SpO2 also showed strong influences on outcome prediction, with clear patterns in their SHAP value distributions.

To explore potential relationships among patient characteristics, we conducted a hierarchical clustering analysis based on these features. The resulting heatmap (Figure 5C) revealed distinct patient clusters, highlighting the heterogeneity within the S-AKI population. The clustering pattern suggested complex interactions between clinical parameters and outcomes.

We further investigated feature interactions through detailed SHAP dependency plots. The relationship between maximum lactate levels and SHAP values (Figure 5D) showed a positive correlation, with higher lactate levels associated with higher SHAP values; notably, early CRRT interventions were more common in patients with elevated lactate levels. Age demonstrated a positive correlation with SHAP values (Figure 5E), though CRRT timing was evenly distributed across age groups. Minimum SpO2 (Figure 5F) exhibited a negative correlation with SHAP values, with early CRRT being more frequent in cases of severe hypoxemia. SOFA scores (Figure 5G) showed a notable increase in SHAP values at higher severity levels (7–9), where early CRRT interventions were more commonly observed, suggesting disease severity significantly influenced CRRT timing decisions.

## Discussion

This study offers valuable insights into the optimal timing of CRRT initiation in patients with S-AKI, providing important findings that may influence clinical decision-making. First, while late CRRT initiation is associated with improved survival, it comes at the cost of increased healthcare resource utilization. Second, we developed a robust GBM model to predict overall survival, highlighting that prognosis in S-AKI patients undergoing CRRT is shaped by multiple organ system factors, rather than just renal parameters. Third, the timing of CRRT initiation has minimal impact on survival prediction compared to other clinical parameters, suggesting that the focus on optimal CRRT timing may be less critical than previously thought.

The timing of CRRT initiation in S-AKI has been a contentious issue, with various studies offering conflicting recommendations. Some research advocates for early CRRT initiation, suggesting it may improve 28-day survival rates (11), enhance SOFA scores, and expedite renal function recovery (12). However, other studies, including those by Barbar et al. (15) and Gaudry et al. (16, 17), found no significant survival benefit from early CRRT initiation and even reported higher rates of catheter-related bloodstream infections in these groups. The inconsistency in these findings likely stems from differences in study design, patient populations, and definitions of “early” versus “late” initiation.

Our study contributes significant insights to the ongoing debate regarding optimal CRRT timing in S-AKI through analysis of large-scale datasets with robust statistical methodology. After propensity score matching to balance baseline characteristics, we observed that late CRRT initiation was associated with increased survival probability. However, this survival advantage came with longer hospital and ICU stays, as well as extended duration of mechanical ventilation, suggesting a complex trade-off between survival benefits and healthcare resource utilization.

In our subgroup analyses stratified by disease severity, we observed consistent trends favoring late CRRT initiation across all SOFA score categories. This survival advantage was most pronounced in patients with high SOFA scores (>15, *p* = 0.058), where early CRRT was associated with notably lower survival rates by day 28. Similar patterns were observed in both medium (11–15, *p* = 0.055) and low SOFA groups (≤10, *p* = 0.063), with late CRRT consistently showing higher survival probabilities throughout the follow-up period.

When stratified by creatinine levels, we found a significant survival advantage for late CRRT in patients with low creatinine levels (≤3 mg/dL, *p* < 0.0001), while no significant differences were observed in medium (3–5 mg/dL, *p* = 0.57) or high creatinine groups (>5 mg/dL, *p* = 0.18). These findings challenge the intuitive assumption that earlier intervention would yield better outcomes, particularly in patients with severe kidney injury. Instead, our results suggest that the optimal timing of CRRT initiation should be personalized, with particular attention to overall disease severity rather than relying solely on renal parameters (29).

It is crucial to recognize that initiation time is just one of many factors influencing the prognosis of patients with S-AKI (30). By integrating multidimensional patient information, including vital signs, laboratory tests, demographics, and comprehensive severity scores, we developed various machine learning models for prognostic prediction. Particularly, the GBM model provided robust survival predictions. The SHAP value analysis revealed that maximum lactate levels, age, and minimum SpO2 were the most influential predictors of survival, followed by SOFA score and maximum PTT. Notably, while the interval from AKI onset to CRRT initiation was included in our analysis, it showed the lowest SHAP value among all evaluated features, suggesting that CRRT timing plays a less critical role in determining patient outcomes compared to markers of systemic illness severity and organ dysfunction.

Our machine learning analysis identified maximum lactate level as the strongest predictor of mortality in S-AKI patients requiring CRRT, followed by age. The paramount importance of lactate aligns with its well-established role as a marker of tissue hypoperfusion and cellular dysfunction in sepsis. In the context of S-AKI, elevated lactate levels not only reflect compromised macro-hemodynamics but also indicate profound cellular metabolic derangement and mitochondrial dysfunction. This finding suggests that the degree of tissue hypoperfusion and metabolic crisis, rather than traditional renal parameters, may be the primary determinant of survival in these patients. The significant impact of age as the second most important predictor likely reflects decreased physiological reserve and impaired ability to recover from severe systemic illness in older patients. Together, these findings emphasize that the prognosis of patients with S-AKI is more strongly influenced by markers of systemic illness severity and host factors than by parameters directly related to kidney injury or CRRT timing.

In terms of clinical implementation, our GBM model has several practical considerations. While the model demonstrates robust predictive performance, it requires standardized data input from electronic health records and has a long processing time. Although SHAP analysis enhances interpretability by identifying key predictive features like maximum lactate and age, the model’s complexity presents challenges for routine clinical use. Future validation across diverse healthcare settings and prospective studies are needed to evaluate the model’s real-world clinical utility.

Furthermore, the clustering analysis revealed distinct patient subgroups based on clinical features, with patients receiving later CRRT initiation tending to cluster together. This suggests that the timing of CRRT may be a proxy for overall clinical status rather than an independent determinant of outcomes. It underscores the importance of personalized decision-making based on a comprehensive assessment of patient condition rather than adhering to a one-size-fits-all approach to CRRT timing (31).

Our study has several limitations that warrant consideration. First, as an observational study using retrospective data, it is subject to potential confounding factors and selection bias, despite our rigorous propensity score matching approach. Second, while we used the eICU database as external validation, which strengthens our findings, the generalizability of our results to all healthcare settings requires further investigation. Additionally, our analysis was limited to the variables available in these databases, and there might be other important factors not captured in our models.

In conclusion, our study demonstrates that later initiation of CRRT in patients with S-AKI is associated with improved survival, though this benefit comes with increased healthcare resource utilization. Our machine learning analysis reveals that systemic illness markers, particularly maximum lactate levels and age, are the strongest predictors of mortality, while CRRT timing plays a less crucial role than previously thought. These findings suggest that clinical decision-making regarding CRRT initiation should focus more on overall illness severity and patient characteristics rather than adhering to strict timing protocols. Future prospective, multicenter studies are needed to validate these findings and develop more personalized approaches to CRRT initiation in patients with S-AKI.

## Data Availability

Publicly available datasets were analyzed in this study. This data can be found at: https://physionet.org/content/mimiciv/3.0/.

## References

[1] Singer M Deutschman CS Seymour CW Shankar-Hari M Annane D Bauer M. The third international consensus definitions for sepsis and septic shock (Sepsis-3). JAMA. (2016) 315:801–10. doi: 10.1001/jama.2016.0287, PMID: 26903338 PMC4968574

[2] Bellomo R Kellum JA Ronco C Wald R Martensson J Maiden M. Acute kidney injury in sepsis. Intensive Care Med. (2017) 43:816–28. doi: 10.1007/s00134-017-4755-7, PMID: 28364303

[3] Kellum JA Chawla LS Keener C Singbartl K Palevsky PM Pike FL. The effects of alternative resuscitation strategies on acute kidney injury in patients with septic shock. Am J Respir Crit Care Med. (2016) 193:281–7. doi: 10.1164/rccm.201505-0995OC, PMID: 26398704 PMC4803059

[4] Wald R Quinn RR Luo J Li P Scales DC Mamdani MM. Chronic dialysis and death among survivors of acute kidney injury requiring dialysis. JAMA. (2009) 302:1179–85. doi: 10.1001/jama.2009.1322, PMID: 19755696

[5] Bagshaw SM Uchino S Bellomo R Morimatsu H Morgera S Schetz M. Septic acute kidney injury in critically ill patients: clinical characteristics and outcomes. Clin J Am Soc Nephrol. (2007) 2:431–9. doi: 10.2215/CJN.03681106, PMID: 17699448

[6] Uchino S Kellum JA Bellomo R Doig GS Morimatsu H Morgera S. Acute renal failure in critically ill patients: a multinational, multicenter study. JAMA. (2005) 294:813–8. doi: 10.1001/jama.294.7.813, PMID: 16106006

[7] Bagshaw SM Lapinsky S Dial S Arabi Y Dodek P Wood G. Acute kidney injury in septic shock: clinical outcomes and impact of duration of hypotension prior to initiation of antimicrobial therapy. Intensive Care Med. (2009) 35:871–81. doi: 10.1007/s00134-008-1367-2, PMID: 19066848

[8] Ronco C Ricci Z. Renal replacement therapies: physiological review. Intensive Care Med. (2008) 34:2139–46. doi: 10.1007/s00134-008-1258-6, PMID: 18791697

[9] Fathima N Kashif T Janapala RN Jayaraj JS Qaseem A. Single-best choice between intermittent versus continuous renal replacement therapy: a review. Cureus. (2019) 11:e5558. doi: 10.7759/cureus.5558, PMID: 31695978 PMC6820322

[10] Hoste EA Bagshaw SM Bellomo R Cely CM Colman R Cruz DN. Epidemiology of acute kidney injury in critically ill patients: the multinational AKI-EPI study. Intensive Care Med. (2015) 41:1411–23. doi: 10.1007/s00134-015-3934-7, PMID: 26162677

[11] Oh HJ Shin DH Lee MJ Koo HM Doh FM Kim HR. Early initiation of continuous renal replacement therapy improves patient survival in severe progressive septic acute kidney injury. J Crit Care. (2012) 27:743.e9. doi: 10.1016/j.jcrc.2012.08.001, PMID: 23084133

[12] Fan Y Chen L Jiang S Huang Y Leng Y Gao C. Timely renal replacement therapy linked to better outcome in patients with sepsis-associated acute kidney injury. J Intensive Med. (2022) 2:173–82. doi: 10.1016/j.jointm.2022.03.004, PMID: 36789016 PMC9923993

[13] An N Chen R Bai Y Xu M. Efficacy and prognosis of continuous renal replacement therapy at different times in the treatment of patients with sepsis-induced acute kidney injury. Am J Transl Res. (2021) 13:7124–31. PMID: 34306472 PMC8290701

[14] Zarbock A Kellum JA Schmidt C van Aken H Wempe C Pavenstädt H. Effect of early vs delayed initiation of renal replacement therapy on mortality in critically ill patients with acute kidney injury: the ELAIN randomized clinical trial. JAMA. (2016) 315:2190–9. doi: 10.1001/jama.2016.5828, PMID: 27209269

[15] Barbar SD Clere-Jehl R Bourredjem A Hernu R Montini F Bruyère R. Timing of renal-replacement therapy in patients with acute kidney injury and sepsis. N Engl J Med. (2018) 379:1431–42. doi: 10.1056/NEJMoa1803213, PMID: 30304656

[16] Gaudry S Hajage D Schortgen F Martin-Lefevre L Pons B Boulet E. Initiation strategies for renal-replacement therapy in the intensive care unit. N Engl J Med. (2016) 375:122–33. doi: 10.1056/NEJMoa1603017, PMID: 27181456

[17] Gaudry S Hajage D Benichou N Chaïbi K Barbar S Zarbock A. Delayed versus early initiation of renal replacement therapy for severe acute kidney injury: a systematic review and individual patient data meta-analysis of randomised clinical trials. Lancet. (2020) 395:1506–15. doi: 10.1016/S0140-6736(20)30531-6, PMID: 32334654

[18] Li X Liu C Mao Z Li Q Zhou F. Timing of renal replacement therapy initiation for acute kidney injury in critically ill patients: a systematic review of randomized clinical trials with meta-analysis and trial sequential analysis. Crit Care. (2021) 25:15. doi: 10.1186/s13054-020-03451-y, PMID: 33407756 PMC7789484

[19] Yoo KD Noh J Bae W An JN Oh HJ Rhee H. Predicting outcomes of continuous renal replacement therapy using body composition monitoring: a deep-learning approach. Sci Rep. (2023) 13:4605. doi: 10.1038/s41598-023-30074-4, PMID: 36944678 PMC10030803

[20] An JN Kim SG Song YR. When and why to start continuous renal replacement therapy in critically ill patients with acute kidney injury. Kidney Res Clin Pract. (2021) 40:566–77. doi: 10.23876/j.krcp.21.043, PMID: 34781642 PMC8685358

[21] Johnson AEW Bulgarelli L Shen L Gayles A Shammout A Horng S. MIMIC-IV, a freely accessible electronic health record dataset. Sci Data. (2023) 10:1. doi: 10.1038/s41597-022-01899-x, PMID: 36596836 PMC9810617

[22] Disease K. Improving global outcomes CKDWG. KDIGO 2024 clinical practice guideline for the evaluation and Management of Chronic Kidney Disease. Kidney Int. (2024) 105:S117–314. doi: 10.1016/j.kint.2023.10.018, PMID: 38490803

[23] Austin PC. An introduction to propensity score methods for reducing the effects of confounding in observational studies. Multivariate Behav Res. (2011) 46:399–424. doi: 10.1080/00273171.2011.568786, PMID: 21818162 PMC3144483

[24] Ho D Imai K King G Stuart EA. Match it: nonparametric Preprocessing for parametric causal inference. J Stat Softw. (2011) 42:1–28. doi: 10.18637/jss.v042.i08, PMID: 36539274

[25] Wen X Bai S Fang Z Zhu W. Integrated pan-cancer and sc RNA-seq analyses identify a prognostic coagulation-related gene signature associated with tumor microenvironment in lower-grade glioma. Discov Oncol. (2024) 15:256. doi: 10.1007/s12672-024-01114-w, PMID: 38955935 PMC11219639

[26] Liu Z Liu L Weng S Guo C Dang Q Xu H. Machine learning-based integration develops an immune-derived lnc RNA signature for improving outcomes in colorectal cancer. Nat Commun. (2022) 13:816. doi: 10.1038/s41467-022-28421-6, PMID: 35145098 PMC8831564

[27] Heagerty PJ Lumley T Pepe MS. Time-dependent ROC curves for censored survival data and a diagnostic marker. Biometrics. (2000) 56:337–44. doi: 10.1111/j.0006-341x.2000.00337.x, PMID: 10877287

[28] Molnar C Casalicchio G Bischl B. Iml: An R package for interpretable machine learning. J Open Source Software. (2018) 3:786. doi: 10.21105/joss.00786

[29] Agapito Fonseca J Gameiro J Marques F Lopes JA. Timing of initiation of renal replacement therapy in sepsis-associated acute kidney injury. J Clin Med. (2020) 9:1413. doi: 10.3390/jcm9051413, PMID: 32397637 PMC7290350

[30] Zarbock A Gomez H Kellum JA. Sepsis-induced acute kidney injury revisited: pathophysiology, prevention and future therapies. Curr Opin Crit Care. (2014) 20:588–95. doi: 10.1097/MCC.0000000000000153, PMID: 25320909 PMC4495653

[31] Yoon BR Leem AY Park MS Kim YS Chung KS. Optimal timing of initiating continuous renal replacement therapy in septic shock patients with acute kidney injury. Sci Rep. (2019) 9:11981. doi: 10.1038/s41598-019-48418-4, PMID: 31427640 PMC6700095

