# Supplementary material for: Machine learning prediction models for mortality risk in sepsis-associated acute kidney injury: evaluating early versus late CRRT initiation
Source: Front Med (Lausanne). 2025 Jan 22;11:1483710. doi: 10.3389/fmed.2024.1483710 (PMC11794530; doi:10.3389/fmed.2024.1483710)
Supplement: Supplementary file 1 [file Data_Sheet_1.docx]

Supplementary Material

# Supplementary Figures and Tables

## Supplementary Figures

**
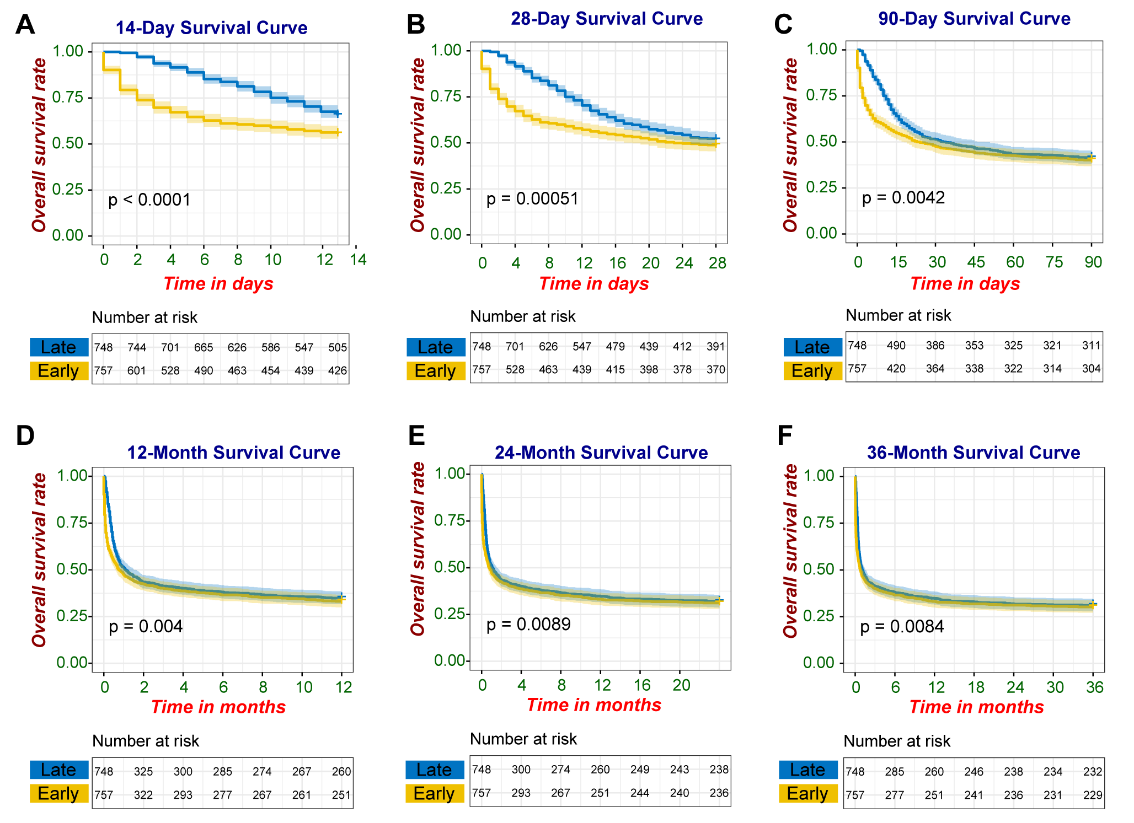
**

**Supplementary Figure 1. Survival Analysis of Early versus Late CRRT Initiation Groups in the Original Cohort.** Kaplan-Meier survival curves comparing short-term and long-term outcomes: (A) 14-day, (B) 28-day, (C) 90-day, (D) 12-month, (E) 24-month, and (F) 36-month survival. Blue and yellow curves represent late (n=748) and early (n=757) CRRT initiation groups, respectively. P-values indicate log-rank test results between groups. Gray shading represents 95% confidence intervals. Numbers at risk are displayed below each curve at corresponding time points.


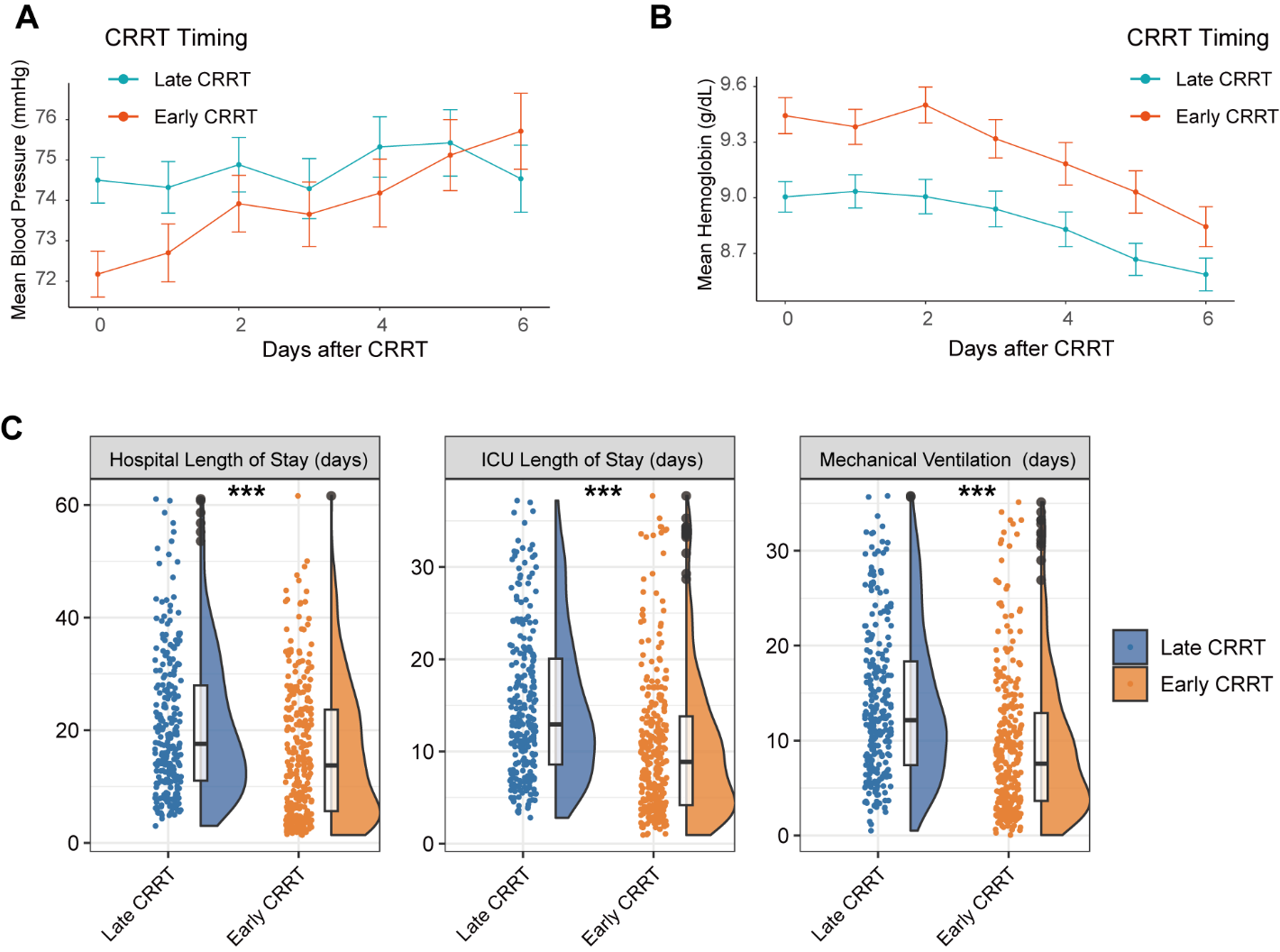


**Supplementary Figure 2. Clinical Parameter Trends and Outcome Comparisons Between Early and Late CRRT Groups.** (A) Mean arterial pressure (mmHg) trends over 6 days following CRRT initiation. (B) Temporal changes in hemoglobin levels (g/dL) over 6 days post-CRRT initiation. Error bars represent standard error of the mean. (C) Outcome comparisons using violin plots with embedded box plots showing the distribution of hospital length of stay, ICU length of stay, and mechanical ventilation duration (all in days). Box plots display median and interquartile ranges with individual data points overlaid. Violin plots demonstrate probability density distribution. Blue and orange represent Late and Early CRRT groups, respectively. ***p < 0.001 for all comparisons in panel C.

## Supplementary table

**Table 1. Baseline Characteristics of Patients Before Propensity Score Matching**

| **Variables (mean ± SD)** | **Late CRRT** | **Early CRRT** | **P value** |
| --- | --- | --- | --- |
| **Number** | 516 | 517 |  |
| **Age** | 64.31 (14.83) | 63.52 (14.54) | 0.385 |
| **SOFA score** | 9.11 (3.70) | 10.58 (3.84) | <0.001 |
| **Maximum Creatinine** | 2.88 (3.97) | 3.71 (2.44) | <0.001 |
| **Minimum Platelets** | 157.72 (108.59) | 146.12 (100.51) | 0.075 |
| **Minimum MBP** | 54.13 (14.29) | 51.93 (13.80) | 0.012 |
| **Maximum Potassium** | 4.86 (0.93) | 5.18 (1.08) | <0.001 |
| **Minimum Bicarbonate** | 19.36 (5.56) | 17.19 (5.49) | <0.001 |
| **Maximum INR** | 1.98 (1.63) | 2.32 (1.93) | 0.002 |

**Table 2. Baseline Characteristics of Patients After Propensity Score Matching**

| **Variables (mean ± SD)** | **Late CRRT** | **Early CRRT** | **P value** |
| --- | --- | --- | --- |
| **Number** | 296 | 296 |  |
| **Age** | 65.59 (14.03) | 63.62 (14.60) | 0.096 |
| **SOFA score** | 9.92 (3.20) | 10.19 (3.32) | 0.314 |
| **Maximum Creatinine** | 2.72 (1.65) | 2.84 (1.65) | 0.388 |
| **Minimum Platelets** | 149.74 (109.20) | 141.39 (98.00) | 0.328 |
| **Minimum MBP** | 54.04 (10.56) | 53.71 (10.68) | 0.707 |
| **Maximum Potassium** | 4.86 (0.78) | 4.93 (0.82) | 0.309 |
| **Minimum Bicarbonate** | 18.61 (4.62) | 18.19 (4.90) | 0.281 |
| **Maximum INR** | 1.78 (0.82) | 1.91 (0.82) | 0.054 |

**Table 3. CRRT Comparison Table**

| **Parameter** | **Early CRRT** | **Late CRRT** | **Estimate** | **F value** | **P value** |
| --- | --- | --- | --- | --- | --- |
| Mean Blood Pressure (mmHg) | 73.7 ± 11.1 | 74.7 ± 10.6 | -1.593 | 4.095 | 0.043* |
| Time effect (per day) | - | - | -0.029 | 0.203 | 0.652 |
| Hemoglobin (g/dL) | 9.28 ± 1.51 | 8.90 ± 1.37 | 0.326 | 8.473 | 0.004** |
| Time effect (per day) | - | - | -0.091 | 169.034 | <0.001*** |
| Had infection | 240/256 | 259/261 |  |  | 0.001573 |
